# The Allosteric Regulation of Β-Ureidopropionase Depends on Fine-Tuned Stability of Active-Site Loops and Subunit Interfaces

**DOI:** 10.3390/biom13121763

**Published:** 2023-12-08

**Authors:** Daniela Cederfelt, Dilip Badgujar, Ayan Au Musse, Bernhard Lohkamp, U. Helena Danielson, Doreen Dobritzsch

**Affiliations:** 1 Department of Chemistry—BMC, Uppsala University, 751 23 Uppsala, Sweden; daniela.cederfelt@kemi.uu.se (D.C.); dilip.badgujar@icm.uu.se (D.B.); helena.danielson@kemi.uu.se (U.H.D.); 2 Department of Cell and Molecular Biology, Uppsala University, 751 23 Uppsala, Sweden; 3 School of Science and Technology, Örebro University, 701 82 Örebro, Sweden; 4 Department of Medical Biochemistry and Biophysics, Karolinska Institute, 171 77 Stockholm, Sweden; bernhard.lohkamp@ki.se

**Keywords:** pyrimidine degradation, 5-fluorouracil metabolism, amidohydrolase, allosteric regulation, cryo-electron microscopy

## Abstract

The activity of β-ureidopropionase, which catalyses the last step in the degradation of uracil, thymine, and analogous antimetabolites, is cooperatively regulated by the substrate and product of the reaction. This involves shifts in the equilibrium of the oligomeric states of the enzyme, but how these are achieved and result in changes in enzyme catalytic competence has yet to be determined. Here, the regulation of human β-ureidopropionase was further explored via site-directed mutagenesis, inhibition studies, and cryo-electron microscopy. The active-site residue E207, as well as H173 and H307 located at the dimer–dimer interface, are shown to play crucial roles in enzyme activation. Dimer association to larger assemblies requires closure of active-site loops, which positions the catalytically crucial E207 stably in the active site. H173 and H307 likely respond to ligand-induced changes in their environment with changes in their protonation states, which fine-tunes the active-site loop stability and the strength of dimer–dimer interfaces and explains the previously observed pH influence on the oligomer equilibrium. The correlation between substrate analogue structure and effect on enzyme assembly suggests that the ability to favourably interact with F205 may distinguish activators from inhibitors. The cryo-EM structure of human β-ureidopropionase assembly obtained at low pH provides first insights into the architecture of its activated state. and validates our current model of the allosteric regulation mechanism. Closed entrance loop conformations and dimer–dimer interfaces are highly conserved between human and fruit fly enzymes.

## 1. Introduction

The reductive degradation of the pyrimidine nucleobases uracil and thymine occurs in three steps (Figure S1, Supplementary Information), with an initial rate-limiting reduction of uracil and thymine to 5,6-dihydrouracil and 5,6-dihydrothymine catalysed by dihydropyrimidine dehydrogenase (DPD, EC 1.3.1.2), followed by two hydrolysis reactions [1]. The first of those, catalysed by dihydropyrimidinase (DHP, EC 3.5.2.2), opens the ring to generate N-carbamoyl-β-alanine (NCβA, β-ureidopropionate) and N-carbamoyl-β-aminoisobutyrate (NCβAIBA), respectively, from which the second hydrolysis releases the corresponding decarbamoylated β-amino acids, carbon dioxide and ammonia. This third and last step is catalysed by β-ureidopropionase (βUP, EC 3.6.1.6, also called β-alanine synthase or N-carbamoyl-β-alanine amidohydrolase).

The reductive pathway is the primary route of pyrimidine degradation in organisms from all kingdoms of life [1,2], although alternatives such as the oxidative pathway, the *rut* pathway, and the URC pathway exist in certain microorganisms [3,4,5]. It functions in maintaining a balanced supply of pyrimidines serving as building blocks of nucleotides and nucleic acids. Furthermore, uracil degradation yields β-alanine, a modulator of neuropathic pain [6], mediator of inhibitory neurotransmissions (as agonist of GABA and glycine receptors) [7,8], and precursor of pantothenic acid [9] and the neuroprotective dipeptides carnosine and anserine [10,11]. The product of reductive thymine degradation is β-aminoisobutyrate (βAIBA), a muscle-released hormone that induces conversion of white to brown adipose tissue and stimulates secretion of leptin and fatty acid oxidation, thereby contributing to exercise-induced protection from metabolic disease [12,13].

Deficiencies in pyrimidine-degrading enzymes alter the homeostasis of pyrimidines and their metabolites and are associated with neurological abnormalities [14,15,16,17,18,19], although the relationship of βUP deficiency to human phenotypes remains uncertain [20]. Moreover, they are a risk factor for the development of life-threatening side effects in cancer patients receiving 5-fluorouracil (5FU)-based chemotherapy [21]; 5FU and its prodrugs are among the most frequently prescribed drugs for chemotherapy in a variety of cancers, but the rapid metabolic deactivation of ~80% of the administered 5FU dose, unpredictable bioavailability, and metabolite toxicity limit their efficacy [17,21].

βUP in higher eukaryotes are nitrilase-like enzymes [22,23] that use a tetrad of conserved active-site residues for catalysis of the CO–N bond cleavage. In human βUP (HsβUP), these catalytic residues correspond to C233, E119, K196, and E207 [24]. E119 is the catalytic base that, by proton abstraction, enhances the nucleophilicity of C233 for its attack on the carbamoyl carbon of the substrate. The oxyanion of the resulting tetrahedral covalent intermediate is stabilized by K196 [25]. E207 is thought to be the base that activates the water molecule hydrolysing the covalent intermediate [26,27]. Crystallographic studies on *Drosophila melanogaster* βUP (DmβUP) revealed that E207 is not stably positioned in the active site [23,28]. It belongs to one of three flexible loops called entrance loops (EL), which shape the entrance to the active site in the activated enzyme state observed in the presence of the substrate NCβA. Substrate activation of human and rat enzymes is accompanied by a shift in their respective oligomer equilibria towards larger assemblies, which for HsβUP are primarily homooctamers [24,29,30,31]. Correspondingly, enzyme inactivation by increasing concentrations of the βUP reaction product β-alanine is mediated by enzyme dissociation to homodimers. In absence of these ligands, HsβUP exists as a mixture of both types as well as intermediate size oligomers [24]. The entrance loops are freely solvent accessible and disordered in the inactive dimer, but structured and buried in dimer–dimer interfaces upon formation of larger oligomers, and may thus represent the link between enzyme activation and assembly state. Interaction between E207 and the bound substrate could potentially represent the trigger for the necessary enzyme conformational changes.

Interestingly, HsβUP association and dissociation can also be triggered in the absence of its natural ligands by shifts in pH. The enzyme is most responsive to allosteric regulation by its natural ligands at the cytosolic pH of 7.0–7.4, at which it exists at the above mentioned equilibrium of different oligomeric states. At pH 9.0, the enzyme occurs exclusively as dimers, and at pH 5 primarily as octamers [24]. This suggests that substrate activation may depend on changes in charge states of enzyme or ligand functional groups.

In order to identify such groups and further investigate the mechanism of the allosteric regulation of βUP, we targeted four amino acid residues (H173, H307, C233, and E207) for site-directed mutagenesis. The mutant variants were analysed for catalytic activity, and for assembly state by size exclusion chromatography, to determine whether these residues are relevant for the catalytic function of HsβUP or its ability to form higher oligomers. In addition, we studied the effect of analogues of βUP substrates and products on the catalytic and allosteric function of the enzyme to reveal compound structure–activity relationships. Finally, we determined the structure of activated HsβUP using cryo-electron microscopy to gain insight into the oligomer architecture and the mechanism of oligomerization, to answer open questions such as: Do higher oligomers of the human and fruit fly βUP show identical assembly modes? Is the conformation of HsβUP entrance loops also dependent on whether they are buried in dimer–dimer interfaces? And how does binding of substrate in one active site lead to cooperative activation of active sites other subunits?

## 2. Materials and Methods

### 2.1. Structure Modelling and Mutagenesis Target Selection

A homology model of a higher oligomeric state of HsβUP was used to identify residues located at dimer–dimer interfaces with putative function in the allosteric regulation mechanism. This model was originally generated from identical HsβUP subunits whose structure was predicted by the SWISSMODEL server [32] using the crystal structure of βUP from *Drosophila melanogaster* (DmβUP; PDB-IDs 2VHI and 2VHH; [23]) as template. The “Protein interfaces, surfaces and assemblies” service PISA at the European Bioinformatics Institute [33] and CONTACT implemented in the CCP4 suite of programs [34] were used to identify residues located at the dimer–dimer interface. The resulting list of potential mutagenesis targets was shortened to residues that are conserved in orthologs, as this could indicate functional or structural importance. Lastly, we visually inspected the residue interactions at dimer–dimer interfaces of the HsβUP model and DmβUP crystal structures using WinCoot [35], and our focus on residues that can change their charge state with pH narrowed down the list further to the two histidine residues, H173 and H307. Replacement of the subunits in the higher oligomeric state model with those of the (dimeric) HsβUP-T299C crystal structure (PDB-Id: 6FTQ, [24]) or those predicted by AlphaFold [36,37], did not necessitate adjustment of target selections made based on the original homology model. 

### 2.2. Site-Directed Mutagenesis, Protein Expression, and Purification

Site-directed mutagenesis to create βUP variants C233S, H307A, H307N, H173A, H173N, and E207Q was performed using the QuickChange method (Agilent Technologies, Sanya Clara, CA, USA) and the protocol from Maurer et al. [24]. Primers (Thermo Fischer Scientific, Stockholm, Sweden) are listed in Supplementary Table S1. Presence of the mutation was confirmed by DNA sequencing.

Wild-type HSβUP and all variants were expressed and purified according to Maurer et al. [24], with the exception of the following small alterations: *E. coli* cells were disrupted by sonication, the IMAC and desalting steps were performed on the bench top without the help of a chromatographic system, using disposable gravity flow columns. A linear instead of a stepwise gradient was used for elution from the anion exchange column.

### 2.3. Molecular Size Estimation

Analytical gel filtration was performed on a Sephacryl S-200 (16/600 mm, 120 mL) column connected to an Äkta Explorer system (GE Healthcare, Uppsala, Sweden), with 0.6 mg/mL βUP enzyme and buffers 20 mM HEPES, 50 mM NaCl, containing 0, 0.1 mM or 1 mM NCβA (pH 7.4), or 100 mM sodium acetate (pH 5.0).

### 2.4. Thermal Shift Assay

The stability and structural integrity of wild-type βUP and mutated variants were compared using a label-free thermal shift assay performed with a Tycho NT.6 instrument (NanoTemper Technologies GmbH, Munich, Germany). A total of 0.1 mg/mL of the respective βUP variant in 20 mM HEPES, 50 mM NaCl, pH 7.4 were loaded into Tycho standard capillaries (NanoTemper Technologies GmbH, Munich, Germany). The temperature gradient was set to a range from 4 °C to 95 °C.

### 2.5. Enzyme Activity Assay

βUP activity was determined as described in Maurer et al. [24] using a discontinuous assay detecting β-alanine (and other primary amines) following its conversion into a fluorescent isoindole by reaction with ortho-phthalaldehyde (OPA, Sigma Aldrich, St. Louis, MO, USA). Standard βUP assays were performed at 37 °C and pH 6.5, at which the enzyme shows highest catalytic activity. The reaction mixture contained 100 mM MES (pH 6.5), 50 mM NaCl, and 10 µg/mL βUP (total volume = 600 µL). Substrate analogues were added at varying concentrations (0.1–75 mM) for the inhibition studies. Reactions were started by the addition of NCβA after preincubation of both enzyme and substrate solutions at 37 °C. The experiments were performed in duplicates. The obtained initial rates were plotted against substrate concentration. Since HsβUP shows cooperativity towards its substrate, although primarily at cytosolic pH and to a much lesser extent at pH 6.5, the data obtained in the absence of inhibitory compounds were fitted using Equation (1), with n representing the Hill coefficient.

(1) $$v=\frac{V_{{max}^{\times }}{\left[NC\beta A\right]}^{n}}{K_{0.5}^{n}+{\left[NC\beta A\right]}^{n}}$$

Kinetic data obtained in the presence of substrate analogues were analysed for conformance with the classical competitive (2), uncompetitive (3), or non-competitive (4) inhibition modes using the following equations:

(2) $$v=\frac{V_{max}\times [S]}{K_{M}^{app}+[S]},\;\mathrm{with}\;{K_{M}}^{app}=K_{M}(1+\frac{\left[I\right]}{K_{i}})$$

(3) $$v=\frac{V_{max}^{app}\times [S]}{K_{M}^{app}+[S]},\;\mathrm{with}\;V_{max}^{app}=\frac{V_{max}}{1+\frac{\left[I\right]}{\alpha K_{i}}}\;\mathrm{and}\;K_{M}^{app}=\frac{K_{M}}{1+\frac{\left[I\right]}{\alpha K_{i}}}$$

(4) $$v=\frac{V_{max}^{app}\times [S]}{K_{M}+[S]},\;\mathrm{with}\;V_{max}^{app}=\frac{V_{max}}{1+\frac{\left[I\right]}{K_{i}}}$$

### 2.6. Cryo-Electron Microscopy Data Collection and Processing

Two alternative options were pursued to enrich larger oligomeric species of HsβUP in samples used for cryo-EM studies: lowering of the pH to 5.0 and incubation of an inactive mutant variant with NCβA. In both cases, the dominant presence of large oligomeric species in the samples was confirmed by size-exclusion chromatography prior to grid preparation and freezing. The reported cryo-EM structure was obtained from a sample containing 1 mg/mL wild-type HsβUP in 0.1 M sodium acetate, pH 5.0, 50 mM NaCl. QuantiFoil-C R23Cu grids (QuantiFoil Micro Tools GmbH, Großlöbichau, Germany) were glow-discharged for 60 s at 20 mA and 0.38 mBar using an EasiGlow (Ted Pella, Inc., Redding, CA, USA) before 3 µL of sample were pipetted onto the grid, at 100% humidity and 4 °C. After blotting for 4 s, the grids were plunge frozen in liquid ethane using a Vitrobot mark IV (Thermo Fisher Scientific, Waltham, MA, USA). Data were collected on a Titan Krios G3 microscope (Thermo Fisher Scientific, Waltham, MA, USA) at the cryo-EM facility at SciLifeLab (Solna, Sweden). The nominal magnification was 105,000×, corresponding to a pixel size of 0.824 Å, and the acceleration voltage was 300 kV. The movies were collected with a dose rate of 12.889 e^−^/pixel/sec and 2.3 s exposure time for a total dose of 43.661 e^−^/Å^2^. Data collection parameters are summarized in Table 1.

Frames obtained for wildtype HsβUP were aligned, averaged, and dose weighted in cryoSPARC v.4.3.1. [38,39]. Subsequent CTF estimation and downstream processing were also carried out in cryoSPARC. A total of 10,512 movies were recorded, from which 7,205,603 particles were automatically picked. After 2D classification, 603,157 particles were selected for use in 3D refinement. The final map **1**, obtained from a non-uniform refinement, had a resolution varying between 3.0 and >6 Å. A new 3D classification was performed, applying masks for the flexible, low-resolution dimers at both ends of the octamer model. The non-uniform refinement [39] using the octamer 3D class yielded map **2** with ~4 Å resolution across the whole map.

Samples of βUP-C233S at 1 mg/mL in 20 mM HEPES pH 7.0, 50 mM NaCl, and 2 mM NCβA were vitrified on holey carbon grids using the same procedure and material as described above. Data were collected on Titan Krios G3 microscope (Thermo Fisher Scientific, Waltham, MA, USA) at the SciLifeLab cryoEM facility (Solna, Sweden). 

Processing of the data obtained for HsβUP-C233S was performed as described above. A total of 11,066 movies were recorded, from which 11,256,089 particles were picked. After 2D classification, 304,440 particles were selected and used for 3D refinement. The 3D classification showed only dimeric particles, and the final map yielded from a non-uniform refinement had a resolution of ~6 Å. As a crystal structure is available for dimeric species of HsβUP, no further data evaluation and model building was performed.

### 2.7. Model Building and Refinement

Map **1** was used to model the centrally located homotetramer, and map **2** to model the two dimers extending the tetramer at both ends to an octamer. Model fitting, morphing, and manual model building was performed in WinCoot [35], with the AlphaFold-predicted structure of HsβUP as a starting model rather than the HsβUP-T299C crystal structure (PDB-Id: 6FTQ, [24]). Both models are largely identical, except for the entrance loops and N-terminal extension. The crystal structure lacks about half of the latter, and the entrance loops adopt a non-native conformation due to the T299C exchange, which leads to the formation of an intramolecular disulphide bridge involving C299. In the AlphaFold model, the entrance loops and N-terminal extension show high similarity to the DmβUP crystal structure. Phenix [40] and the implemented MolProbity [41] function were used for model refinement and structure validation, respectively. Data statistics are summarized in Table 1. The atomic coordinates and cryo-EM map **1** were deposited in the Protein Data Bank (PDB, accession code 8PT4) and Electron Microscopy Data Base (EMDB, Id: 17867). Protein structure figures were generated with ChimeraX [42] and PyMol [43].

## 3. Results and Discussion

### 3.1. Site-Directed Mutagenesis

**Figure 1:**
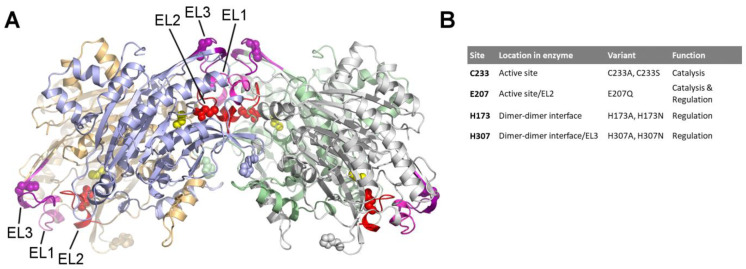
Site-directed mutagenesis of HsβUP. (**A**) Homology model of a HsβUP homotetramer (cartoon). Each subunit is coloured differently, with the exception of entrance loops (EL) 1, 2, and 3, which are depicted in light magenta, red, and purple, respectively. The four residues targeted by site-directed mutagenesis are shown as space-fill models in all four subunits. C233, E207, and H307 are coloured yellow, red, and purple to emphasize their location in the active sites, EL2 and EL3, respectively, whereas H173 is coloured as the subunit it belongs to. (**B**) Table listing details of the mutation sites and created variants.

Site-directed mutagenesis was performed to gain further insights into the relationship between the HsβUP oligomerization state and catalytic activity, and to pinpoint the enzyme residues that play a role in the allosteric mechanism. Since the allosteric regulation of the enzyme by its ligands can be mimicked by shifts in pH, we were specifically looking for conserved residues located at or near the dimer–dimer interface that could alter their charge state within the pH 5–9 range. We identified two histidines, H173 and H307, that fit the requirements by analysis of a HsβUP homology model generated with SwissModel [32] based on the crystal structure of the DmβUP homooctamer [23] (Figure 1). The crystal structure of a T299C mutant of HsβUP [24] could not, due to its dimeric state, provide precise observations of residue interactions at the dimer–dimer interface, but the close resemblance between the surface features of model and experimental structure supported the target selections made based on the homology model.

**Figure 2:**
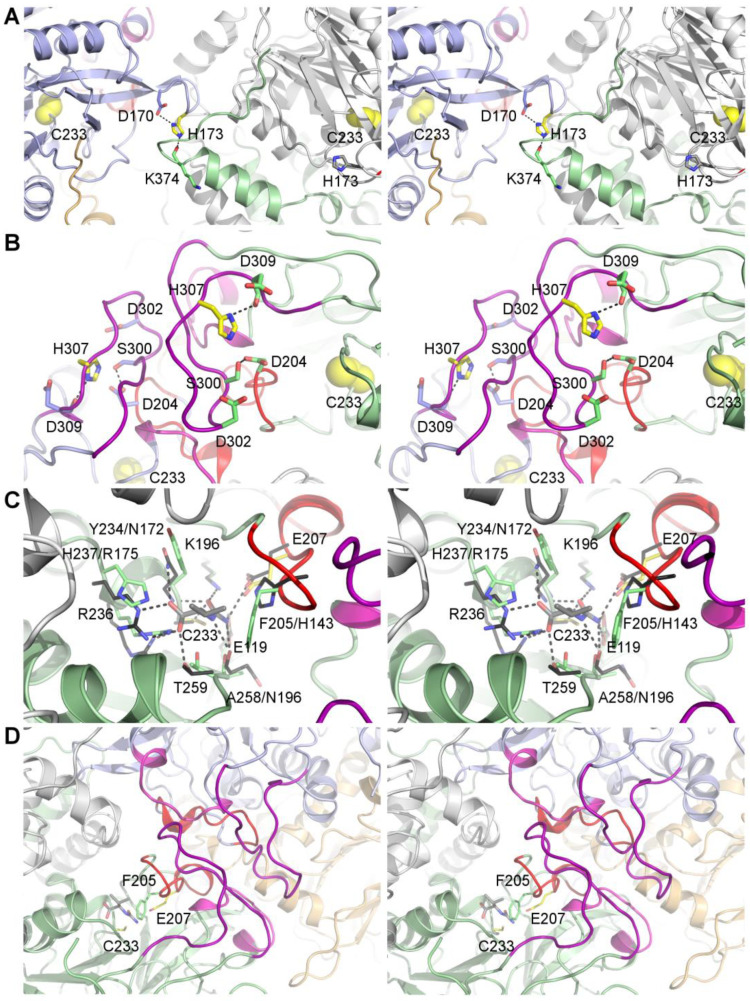
Environments of the mutation site. Each pair of images creates a wall-eye stereo view providing a 3D impression. The HsβUP homology model is shown as a cartoon with different colours for distinct subunits and entrance loops (colours as in Figure 1). Residues targeted for mutagenesis are shown as sticks with carbon atoms in yellow in the two subunits in which they are placed close to the dimer–dimer interface, all other residues have carbon atoms in the colour of the subunit they belong to. In (**A**,**B**), the location of the active site is highlighted by the space-fill model of C233 (yellow). Black dashed lines indicate hydrogen bonds. (**A**) Interactions of H173. (**B**) Interactions of H307. (**C**) Environment of C233 and E207. To visualize their approximate position relative to the substrate during the catalytic cycle, the crystal structure of the homologous *Agrobacterium* sp. N-carbamoyl-D-amino acid amidohydrolases (DCase) double mutant C171A/V236A (PDB ID: 1uf7, unpublished) in complex with its substrate N-carbamoyl-D-valine (carbon atoms in dark grey) was superimposed. DCase residues involved in ligand binding are shown as sticks in black. If the residue type is not conserved, labels state the DCase residue after the corresponding HsβUP residue. (**D**) Zoomed-out view of (**C**), emphasizing the position of C233 and E207 relative to the entrance loops at the dimer–dimer interface. The DCase substrate is shown together with the HsβUP residues C233, E207, and F205.

H173 is located in a loop connecting two long β-strands of the β-sandwich core fold (Figure 1 and Figure 2). The corresponding loop of DmβUP adopts the same conformations in subunits that are, and those that are not, involved in dimer–dimer interface formation, and hence does not seem to alter its backbone structure upon interface formation. This may in part be due to the hydrogen bond its side chain forms with another loop residue, D170 (Figure 2). Upon burial in dimer–dimer interfaces, H173 forms another hydrogen bond with the main chain carbonyl group of K374 from the opposite dimer. K374 is the residue directly following the C-terminal helix that largely mediates a “mutual hugging” of the subunits forming the dimer, and is thus more tightly associated with the partner subunit than the polypeptide chain it belongs to. Simultaneous hydrogen bonding with both D170 and K374 at physiological pH, at which the D170 side chain can be expected to be deprotonated, should only be possible if H173 is protonated (Figure S2). Deprotonation of H173 occurring with pH increase should sever one of the hydrogen bonds, whereas both can be maintained upon protonation of D170 at low pH, since the absence of any further side chain-mediated bonds gives D170 the ability to act as both hydrogen bond acceptor and donor. Thus, the H173 charge state may contribute to the observed pH-induced shift in oligomer distribution.

H307 belongs to entrance loop 3 (EL3) which adopts a stable structure only upon dimer association to larger oligomers (Figure 1). It contributes to EL3 structuring by forming hydrogen bonds with the backbone carbonyl group of D309 and the side chain of S300, which belong to the same loop (Figure 2). In the DmβUP structure, as well as the AlphaFold [36,37]-derived model of activated HsβUP (which basically combines the subunit fold of the HsβUP-T299C crystal structure with the DmβUP EL conformations and assembly mode), the S300 side chain is sandwiched between the carboxyl groups of D204 from EL2 and D302 from EL3, forcing it to act as a hydrogen bond acceptor towards H307 at neutral and high pH, whereas it can be either donor or acceptor at low pH. As a consequence, the ability of H307 to maintain both hydrogen bond interactions is also pH dependent (Figure S2). At high pH, the deprotonation of the imidazole ring and necessity to act as hydrogen bond donor towards the D309 carbonyl group will sever the interaction with S300. This may contribute to EL3 destabilization and, ultimately, oligomer dissociation.

To test whether H173 and H307 are indeed involved in the allosteric regulation, we mutated both side chains, independently, to alanine and asparagine. Asparagine is largely able to substitute histidine in hydrogen bonding interactions, however, it can only accept but not donate hydrogen bonds with its carboxamide oxygen. In contrast, replacement by alanine will abolish the ability to form side-chain-mediated hydrogen bonds completely.

The βUP mutant E207Q was created to test whether E207 indeed functions as a catalytic base in the HsβUP-catalysed reaction and/or plays a role in the allosteric regulation. In addition, we targeted the active-site nucleophile C233 for replacement by serine (Figure 1 and Figure 2). Together with a previously reported C233A mutant [24], it was aimed at co-crystallization experiments with the substrate NCβA in order to obtain structural information about the activated enzyme state and the substrate binding mode. To date, it is also still unknown whether HsβUP activation is mediated via substrate binding to the active site or yet undiscovered allosteric sites. All enzyme variants were expressed and purified to at least 95% homogeneity following the same procedure as for the wild-type HsβUP.

### 3.2. Characterization of HsβUP Mutants

A discontinuous, fluorometric assay for detection of primary amines was used to measure βUP activity [24]. At pH 6.5, the pH at which the highest catalytic activity is observed, wild-type βUP does not show significant cooperativity towards NCβA, and *k*_cat_ and K_M_ for this substrate were determined to 5.0 s^−1^ mM^−1^ and 101.4 μM, respectively. The HsβUP mutants C233A, C233S, H173A, H173N, H307A, H307N, and E207Q were all inactive (Figure S3), which is consistent with C233 and E207 fulfilling crucial catalytic roles as active-site nucleophile and general base, respectively. However, H173 and H307 are located too distant from the active site to play a direct role in catalysis.

To exclude that the inactivity is caused by detrimental effects of the point mutations on enzyme stability, the inflection temperatures for enzyme thermal unfolding were measured using label-free differential scanning fluorimetry (DSF). For variants H173N, E207Q, H307A, and H307N they are very similar to that of wild-type HsβUP (55.6 °C, Table 2), whereas the inflection temperature of unfolding measured for C233S is 3 °C higher in comparison. Thus, protein stability was retained in the mutant enzymes, which suggests that the lack of catalytic activity of the H173 and H307 variants may result from impairment of the allosteric activation mechanism.

**Figure 3:**
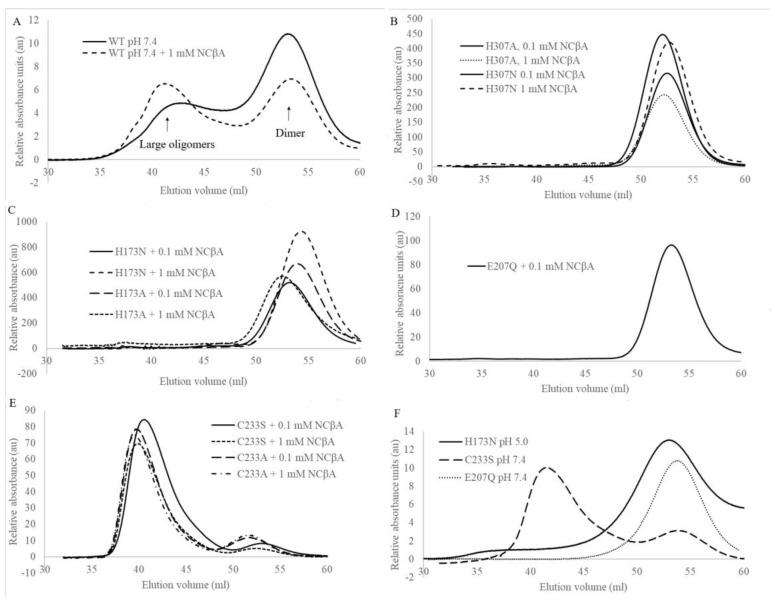
Molecular size distribution of HsβUP variants in the absence and presence of the substrate NCβA. (**A**) Wild-type HsβUP, (**B**) variants H307A and H307N, (**C**) H173A and H173N, (**D**) E207Q, (**E**) C233A and C233S, all at pH 7.4. (**F**) C233S and E207Q at pH 7.4, and H173N at pH 5.0, all in the absence of NCβA. The experiments were performed on a Sephacryl S-200 (16/600 mm, 120 mL) column connected to an Äkta Explorer system (GE Healthcare, Uppsala, Sweden).

Since enzyme activation is accompanied by shifts in oligomer equilibrium, we next determined the dominant oligomeric states of the mutant enzymes in the presence of NCβA using analytical size exclusion chromatography (Figure 3). The experiments were performed at cytosolic pH (7.4), at which the enzyme operates and is most responsive to allosteric regulation. Like wild-type βUP and C233A variant [24], the C233S variant does, at pH 7.4 and in the absence of the natural ligands NCβA and β-alanine, occur as a mixture of different oligomeric assemblies (Figure 3). Addition of 0.1 mM NCβA shifts the oligomer equilibrium so that C233S occurs predominantly as larger oligomers (most likely homooctamer). The chromatograms obtained upon 10-fold increase in NCβA concentration are largely identical, indicating that the lower NCβA concentration is sufficient to fully “activate” the enzyme variant even when the reactive functional group of the active-site nucleophile is lacking. It can be concluded that C233 is not playing a role in the allosteric regulation of HsβUP.

In contrast, H173A, H173N, H307A, and H307N are present exclusively as (inactive) dimers in the absence of NCβA, and remain unresponsive to the βUP substrate even if present at 1 mM concentration. Considering that the side chains of histidine and asparagine are comparable in size and general capacity to engage in hydrogen bonding interactions, but differ in their ability to change charge state near physiological pH or to donate hydrogen bonds to more than one partner with similar geometry, it appears that the HsβUP activation mechanism is dependent on the presence of side chains at positions 173 and 307 that can fine tune their interactions with nearby residues at the dimer–dimer interface and have either just one or both of these properties. Their titration behaviour may, in fact, explain why the shift in oligomer equilibrium caused by allosteric effectors can also be brought about by changes in pH. A physiological relevance of the pH-mediated activation mechanism is, however, questionable. The strict conservation of H173 and H307 may thus instead be linked to H307’s capability to support the structural stabilization of inherently flexible entrance loops in response to reaction substrate and allosteric effector NCβA, and H173’s capability to strengthen subsequently formed subunit interfaces, for which both residues would need to be protonated. Initial EL stabilization likely triggered by substrate binding removes physical obstacles for tight association of HsβUP dimers to higher oligomers, which in turn suppresses loop movement, locks them in active conformation, and stably inserts the catalytic base E207 belonging to EL2 in the active site.

Interestingly, the conservative exchange of E207 by glutamine (E207Q) has the same effects as targeting the conserved interface histidines: HsβUP is rendered unable to form higher oligomers and to become activated by NCβA. This suggests that the ability of E207 to abstract or donate a proton is not only crucial for catalysis, but also for the allosteric regulation. The mechanism of E207’s involvement in the latter remains yet to be uncovered.

### 3.3. Allosteric Effector Studies

To gain further insight into the mechanism by which NCβA and β-alanine trigger EL conformational changes and enzyme association and dissociation, respectively, we performed structure–activity studies with a number of structural analogues of both compounds (Table 3). We selected the commercially available N-carbamoyl-glycine (hydantoic acid), glycyl-glycine, glutaric acid, 2-aminoisobutyric acid, and isobutyric acid. N-carbamoyl-glycine most closely resembles the HsβUP substrate NCβA, as it contains both the carboxyl and N-carbamoyl moieties. Glycyl-glycine and 2-aminoisobutyric acid share the presence of both a carboxyl and amino group with the HsβUP product β-alanine, whereas only the carboxyl group is retained in the remaining two compounds.

**Figure 4:**
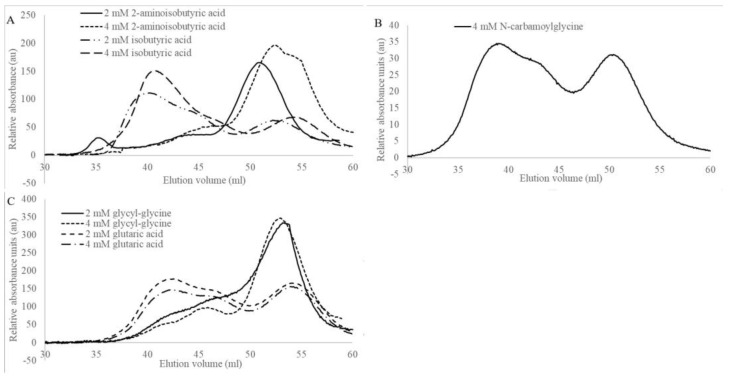
Molecular size distribution of wild-type HsβUP in response to substrate-analogous compounds at pH 7.4; (**A**) 2-Aminoisobutyric acid and isobutyric acid, (**B**) N-carbamoylglycine, and (**C**) glycyl-glycine and glutaric acid. HsβUP occurs mainly as a dimer in the presence of glycyl-glycine and 2-aminoisobutyric acid, as larger assemblies in presence of isobutyric acid, and as a mix of smaller and larger oligomer forms in the presence of glutaric acid and N-carbamoylglycine.

**Figure 5:**
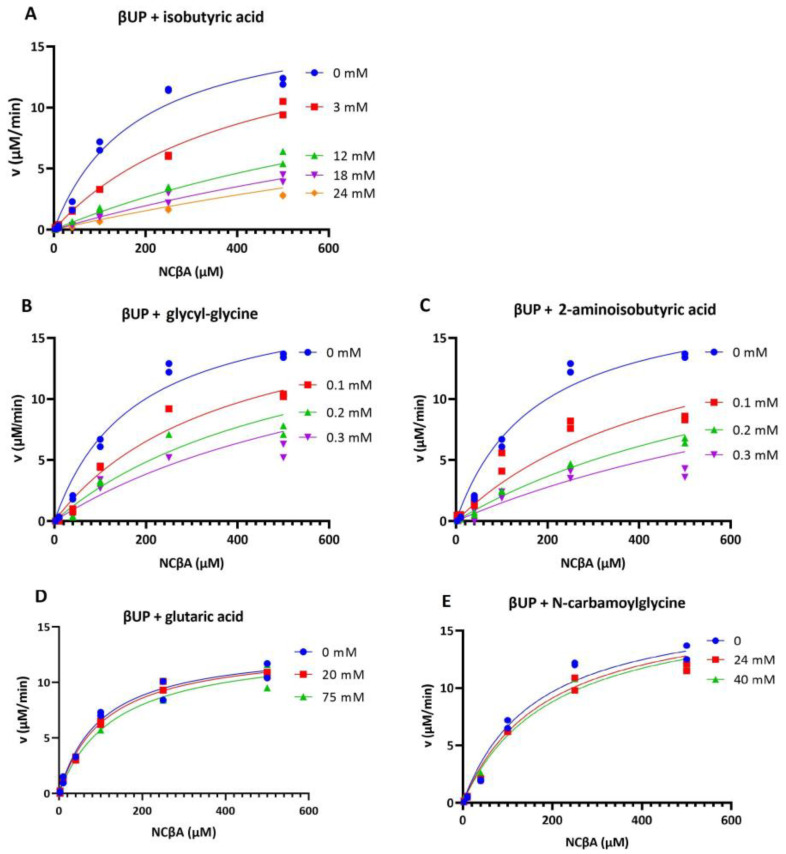
Inhibition of HsβUP by substrate analogues. Enzyme activity with the substrate NCβA was assayed in presence of different concentrations of substrate analogues at 37 °C and pH 6.5, using 10 µg/mL βUP. The measurements were performed in duplicates, with data points for both being shown in the graphs. The effect of isobutyric acid (**A**) on HsβUP activity can be adequately fitted by an equation corresponding to competitive inhibition, whereas the reaction rates measured in the presence of glycyl-glycine (**B**) and 2-aminoisobutyric acid (**C**) are best fitted by applying the equation for non-competitive inhibition (see Figure S4 and Table S2). However, the used equations do not account for the cooperativity of the enzyme induced by the substrate or substrate analogue, and thus the goodness of fit is misleading. The structural similarity of the ligands with reaction substrate and product make competitive inhibition more likely, and thus the data fit corresponding to this inhibition mode is shown here. (**D**) Presence of glutaric acid and (**E**) N-carbamoylglycine did not have any effect on enzyme activity.

Analytical size exclusion experiments performed with glycyl-glycine or 2-aminoisobutyric acid added to the buffer revealed that these compounds have a dissociative effect on wild-type HsβUP, whereas the enzyme mainly occurred as a larger assembly (most likely octamer) in the presence of isobutyric acid (Figure 4). Presence of glutaric acid or N-carbamoylglycine does not shift the oligomer equilibrium when compared to HsβUP in the absence of any ligands. Inhibition studies showed that NCβA conversion by the enzyme is also not affected by the presence of up to 40 mM N-carbamoyl-glycine or 75 mM glutaric acid, which indicates that HsβUP has no or very low affinity for either compound (Figure 5). In contrast, the presence of any of the other three compounds does affect the enzyme-catalysed conversion of NCβA to β-alanine.

Next, we attempted to correlate the structure of the five compounds studied here with the effect exerted on βUP. In our analysis we also included the three substrate analogues, β-alanine and β-aminoisobutyric acid (βAIBA, the product of the βUP-catalysed hydrolysis of N-carbamoyl-β-aminoisobutyrate), which were reported previously [24]. No unambiguous correlation could be found between the number or type of charges and the distinction between enzyme associative or dissociative effect. β-Alanine, glycyl-glycine, and 2-aminoisobutyric acid, the three compounds causing βUP dissociation to dimers, all carry a free amino group likely to be positively charged at physiological pH. However, the pure presence of a positively charged terminus in the ligand is not sufficient to explain this effect, since three of the compounds causing association show the same structural feature. The effect on βUP oligomer equilibrium is also not correlated with the total length/size of the ligand or the spacing between their (usually polar) termini. Interestingly, βUP has (albeit very week) affinity for isobutyric acid, which does not contain an amino, ureido, or amide moiety, which suggests that the carboxyl group all investigated compounds carry is the primary binding determinant. Nevertheless, its presence in N-carbamoylglycine and glutaric acid is not sufficient for the enzyme to bind these two compounds, probably because other functional groups are inappropriately positioned within the ligand to cause favourable, or avoid unfavourable, contacts with active-site residues. This indicates that distribution of polar/charged and non-polar functional groups within the ligands may play a role. In fact, all compounds that contain less than two carbon atoms between the carboxyl carbon and the next polar group have either dissociative effects or are unable to bind to HsβUP (Table 3).

To further analyse the significance of this finding we inspected the active-site architectures of the HsβUP-T299C and DmβUP crystal structures as well as an AlphaFold model of wild-type HsβUP. Since all correspond to the enzyme in ligand-free state, the binding mode of the substrate NCβA, and subsequently also of its analogues, was deduced from the crystal structure of a homologous, mutated N-carbamoyl-D-amino acid amidohydrolase (DCase) bound to its substrate N-carbamoyl-D-valine (Figure 2, PDB accession code 1uf7 (unpublished)). DCase hydrolyses N-carbamoylated α- rather than β-amino acids, using an identical reaction mechanism involving initial nucleophilic attack by an active-site cysteine and a covalent enzyme-substrate intermediate. Furthermore, the identity and location of the residues comprising the catalytic tetrad is conserved. In contrast to the βUP ligands, the DCase ligands contain bulkier hydrophobic groups next to the carboxyl group, which are immediately followed by the carbamoyl moiety. βUP’s discrimination against ligands having a second polar group at equal distance to the carboxyl group may be due to the placement of the F205 side chain, which packs closely against bound ligands upon enzyme activation and ordering of the entrance loops. F205 itself belongs to EL2, which is situated closest to the active-site cavity and thus has to be ordered first, before EL1 and EL3 can follow. Whether favourable hydrophobic interactions can be made with this residue may therefore be what distinguishes association- from dissociation-causing allosteric effectors. Ligands that push F205 to a more remote position in order to avoid interaction of polar groups with its benzyl ring will present an obstacle for the ordering of all three entrance loops, which in turn is a prerequisite for dimer–dimer interface formation. Since the catalytic base E207 is located just two positions downstream on the same loop, any ligand effect on the placement of F205 is likely to also affect the position of E207, thereby determining whether HsβUP is catalytically active or inactive.

Such a mechanism does, however, not fully explain all measured ligand effects. For instance, why does β-alanine have a dissociative and βAIBA an associative effect (Table 3)? In absence of experimental structures showing both compounds bound to the enzyme we can only provide a theoretical analysis. It is possible that the additional methyl group present in βAIBA allows only one particular binding mode that directs the methyl group towards F205, thus effectively providing an anchor point for its side chain while concomitantly shielding the nearby positive charge of the βAIBA amino group, whereas the smaller β-alanine has more freedom to adopt different binding modes in the active-site cavity. Some may place its charged amino group in close proximity to the F205 side chain, causing repulsion and EL opening.

Another open question is why the HsβUP active site is able to accommodate 4-ureidobutyric acid, which, compared to NCβA, is extended by one methylene group, and without causing the ELs to disorder, whereas the enzyme has no measurable affinity for N-carbamoyl-glycine, which is one methylene group shorter than NCβA, but still larger than β-alanine. We manually modelled N-carbamoyl-glycine into the active site, assuming that either the carboxyl or the carbamoyl group interactions with HsβUP residues are equivalent to those observed for N-carbamoyl-valine in the active site of DCase (1uf7, unpublished) (the binding mode leading to identical positioning of the carbamoyl group would correspond to that shown for the DCase substrate in Figure 2C upon removal of the valine side chain). It suggests that the chain length shortening causes either the carbonyl or the carbamoyl group to be placed unfavourably close to Y234. Since Y234 is not located on a flexible entrance loop, this would explain why N-carbamoyl-glycine not only has a dissociate effect, but is also not able to bind to an inactive enzyme state with disordered entrance loops. In contrast, the inability to bind glutaric acid can easily be explained by the fact that its second carboxyl group would need to be inserted between the side chains of E207 and E119, clustering three negative charges together.

To date, it can also not be excluded that the allosteric effect of the natural and analogous ligands is exerted via binding to alternative sites, that may be partially overlapping with the substrate binding site or may become occupied only at higher ligand concentration, and have yet to be discovered. Presence of such alternative sites may explain why enzyme inhibition data obtained with glycyl-glycine and 2-aminoisobutyric acid can be best fitted assuming non-competitive behaviour (Figure 5 and Figure S4). Alternatively, the uncompetitive component of this special case of mixed inhibition mode could also be explained by the intricate regulation mechanism of βUP: inhibitors that are essentially competitive in nature as they occupy the substrate binding site simultaneously act as negative allosteric effectors via their influence on enzyme oligomerization, thus inhibiting a larger number of active sites than they occupy. 

### 3.4. Structural Studies

**Figure 6:**
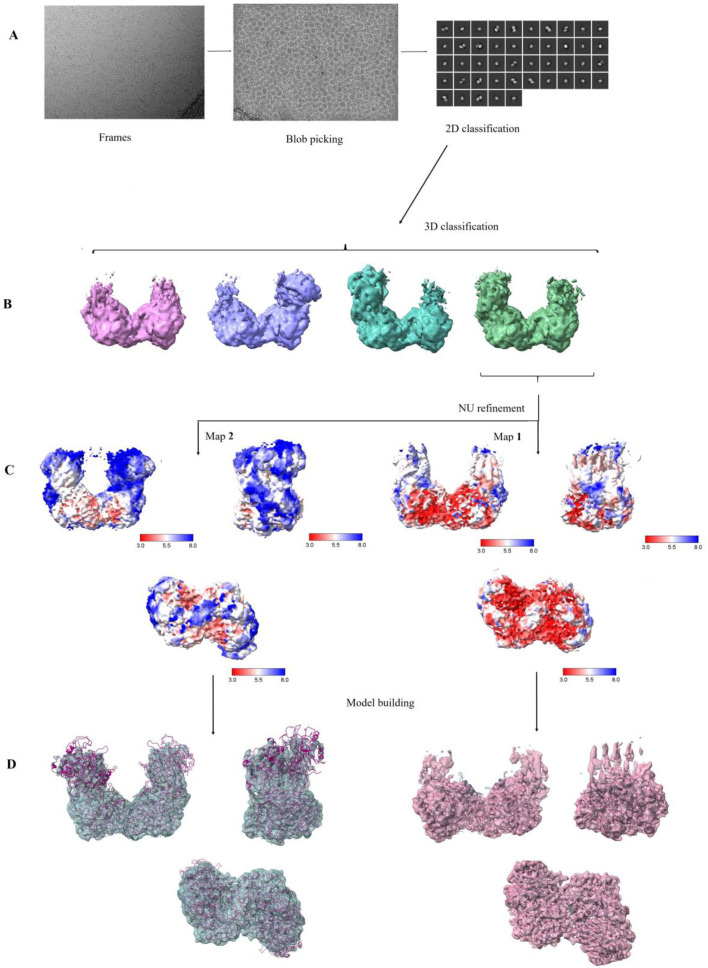
Single particle cryo-EM data processing of HsβUP oligomers assembled at low pH. (**A**) Representative micrograph, blob picking result, and 2D class averages from one independent experiment. (**B**) The 3D classification yielded different oligomer species, i.e., tetramers, hexamers, and octamers. However, no dimeric species were observed. (**C**) Two maps of different resolution were obtained from non-uniform data refinement (here shown in three different orientations). Original data processing yielded map **1**, showing 3–3.5 Å resolution for the 4 central subunits, but much lower resolution for the outer subunits at both ends of the octamer. Map **2** has an overall resolution of ~4 Å, and was obtained after application of masks focusing on the outer subunits. (**D**) On the right, two copies of the crystal structure of dimeric HsβUP are fitted into map **1**, after replacement of non-natively structured loops and addition of missing parts at the N-terminus from an AlphaFold-generated model. Fitting of four dimer copies into map **2** (shown on the left) reveals that the map is incomplete at the periphery, which is most likely an artefact from averaging data obtained for octamers in which the outer dimers assume slightly different positions. The density and structure figures were prepared in Chimera X [42]. The contour levels of the shown cryo-EM maps are 0.25 (**B**), 0.06 (**C**), and 0.075 (**D**). See Figures S5–S7 for GSFSC, local resolution, and correlation coefficient graphs.

In order to reveal the binding sites and modes of NCβA, β-alanine, and other allosteric effectors, we attempted co-crystallization of HsβUP with these compounds. We targeted the active-site nucleophile of HsβUP for site-directed mutagenesis to alanine and serine to obtain mutant variants unable to convert NCβA but capable of forming the activated higher oligomeric state, for co-crystallization experiments with the substrate. Unfortunately, none of the numerous co-crystallization setups resulted in crystals suitable for diffraction experiments, probably because the enzyme solutions were still too heterogeneous despite the achieved shifts in oligomer equilibria to larger states. Thus, we employed single-molecule cryo-electron microscopy (cryo-EM) studies to obtain first insights into the subunit arrangement and EL conformation in activated HsβUP. For this purpose, we prepared grids for the C233S variant with 2 mM NCβA at pH 7.0, and for wild-type HsβUP without any ligands at pH 5.0. Only the latter sample resulted in micrographs that were of sufficient resolution and contained enzyme states larger than the dimer. Nevertheless, 3D classification showed that the sample was heterogeneous, consisting of tetramers, hexamers, and octamers (Figure 6).

Non-uniform refinement of the data for octameric species yielded two maps used for model building. Map **1** has a resolution of ~3.0–3.5 Å for the two central HsβUP dimers, and >6 Å for the “outer” dimers, indicating that the latter region shows more variability. Map **2** was generated using a focus mask for the two “outer” dimers, to increase resolution in these parts. This map has a more or less uniform resolution of ~4–4.5 Å (Figure 6 and Figures S5–S7). The structural model deposited in the PDB does in fact only consist of the two inner-most dimers, as the low resolution map obtained for the outer dimers prohibited a precise modelling of these parts.

#### 3.4.1. Subunit Structure and Dimer Assembly

**Figure 7:**
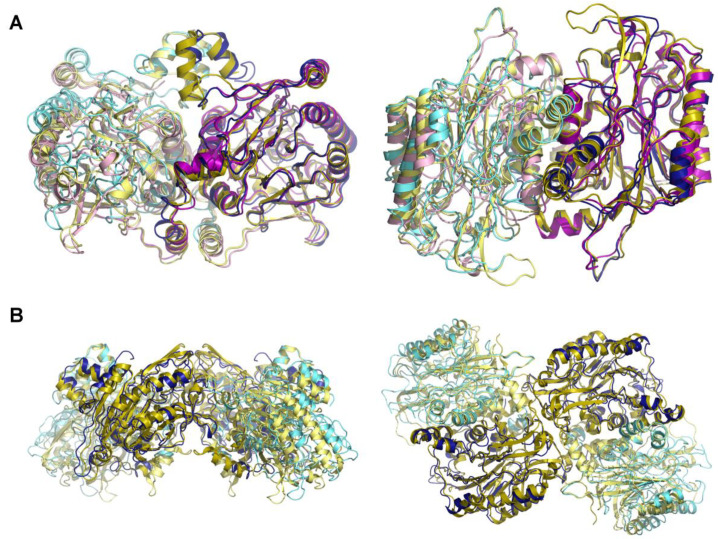
Comparison of the HsβUP cryo-EM structure with the crystal structures of HsβUP-T299C and DmβUP. (**A**) Superimposed homodimers. The view on the left results from a ~90° upward rotation of the view on the right. The subunits are shown in cartoon representation coloured blue and aquamarine for the HsβUP cryo-EM structure, purple and light pink for the HsβUP-T299C crystal structure, and olive and pale yellow for the DmβUP crystal structure. The superposition is based on one of the subunits per dimer (those in darker colours), to highlight the differences in relative position of their mates. (**B**) Superimposed tetramers of HsβUP and DmβUP. Colours and views are as in (**A**). The superposition is based on one of the central subunits, which reveals that the lower part of the outer subunits of HsβUP are shifted out and upwards as compared to the corresponding subunits of DmβUP, giving the HsβUP tetramer a flatter appearance.

Superimposition of the cryo-EM model with the crystal structure of (dimeric) HsβUP-T299C reveals that their core subunit structure consisting of an αββα-sandwich is basically identical (Figure 7). Out of the 384 amino acids comprising the HsβUP subunit, residues 3–300 and 307–368 are visible in the density map. The cryo-EM derived model thus includes the complete N-terminal extension (~60 aa) characteristic of the βUP branch of the nitrilase superfamily [22]. In contrast, the first half of this extension was not resolved in the (crystallographic) electron density map obtained for HsβUP-T299C [24]. The r.m.s.d.s obtained when superimposing the respective individual subunits of both HsβUP models are all ~1.0 Å (Table S3), primarily due to structural deviations of the entrance loops, whose non-native conformation in the crystal structure is caused by an artificial disulphide bridge involving the mutation site (T299C) and affects also neighbouring peripheral stretches of the polypeptide chain. In the cryo-EM structure reported here, the entrance loops as well as the N-terminal extension adopt conformations basically identical to those observed in the crystal structure of DmβUP [23]. EL1 and EL2 are completely ordered, and EL3 partially. The lack of density for at least six residues at the very tip of this loop is indicative of their dynamic mobility. In contrast to the HsβUP (and DmβUP) crystal structure, also about half of the C-terminal helix and the following tail, which are exchanged between monomers and thus important for dimer stabilization, are not visible in the density map.

The subunit surfaces involved in βUP dimerization have been described previously [23,24]. They are well conserved between the human and fruit fly homologs, but a slight tilt of one subunit relative to the other was observed between the crystal structures of HsβUP-T299C and DmβUP, tipping it in the former enzyme in the direction of the N-terminal extension by a few degrees. Resulting displacements of equivalent C_α_-positions are barely noticeable at the subunit interface itself, but become more pronounced towards the outer periphery of the protein molecule. We hypothesized that this tilt could be a consequence of the mutation present in the crystallized HsβUP variant, and the cause of the observed disorder of a large portion of the N-terminal extension. However, in the wild-type HsβUP cryo-EM structure, this tilt of one subunit relative to the other is even more pronounced when compared to a DmβUP dimer, leading to displacements of equivalent C_α_-positions at the outer periphery by up to 10 Å. The resulting rotation of the subunit core towards the four-helix bundle, which is comprised by the two N-terminal helices from both monomers, does, however, not extend to the bundle itself—its coordinates are perfectly superimposable with those of the corresponding DmβUP structure. This shows that the bundle helices do not form a rigid body with the remainder of subunit, but can move independently due to the rather flexible connection between them, i.e., the 12-residue long coil connecting the second bundle helix with the first helix of the core αββα-sandwich domain. This loop does indeed adopt a different conformation at positions 33–35, due to which steric clashes with the bundle helices are avoided.

#### 3.4.2. Assembly of Larger Oligomers and Entrance Loops

**Figure 8:**
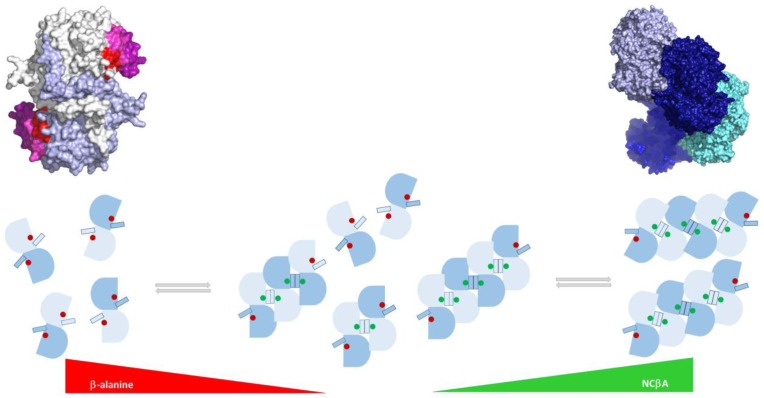
Model of the HsβUP allosteric regulation mechanism. At physiological pH and in the absence of allosteric effectors, HsβUP occurs in an equilibrium of oligomeric states, ranging from dimers to at least octamers. Increasing concentrations of β-alanine or increasing pH causes enzyme dissociation to dimers (left side). These dimers consist of identical subunits (shown in light blue and blue) whose active sites are incomplete due to the disorder of the entrance loops (smaller rectangle) that carry E207. Therefore, dimers are inactive (indicated by a red dot at the location of the active site). In contrast, increasing concentration of NCβA and decrease in pH shifts the equilibrium towards the right end of the figure, with dimers associating first to tetramers, and then (via hexamers or directly) to at least octamers. Substrate binding or pH change cause entrance loop structuring, but formation of dimer–dimer interfaces is required to hold the loops in place. As a consequence, E207 is inserted in the active sites with closed loops, making them catalytically competent (green dot). In the schematic representation of oligomeric species at the bottom, equally coloured subunits make the most extensive contacts at the dimer–dimer interfaces, whereas in the cryo-EM model of the HsβUP octamer shown above, the same colour is used for the two subunits forming a dimer. Note that the octamer has an overall helical turn-like shape that would result in formation of a super-helical structure upon extension by further dimeric units, and that the outward facing active sites located at the ends of the molecule are inactive. In the surface representation of the HsβUP dimer at the top left, one subunit is shown in white and the other in light blue, except for the EL1–3, which are coloured magenta, red, and purple, respectively. The AlphaFold-model of HsβUP was used to assemble this dimer, as it contains the subunit-swapping C-termini as observed in the HsβUP-T299C crystal structure, as well as the complete N-terminal extension as observed in the cryo-EM structure.

The slightly altered orientation of the subunits in the HsβUP dimer compared to the DmβUP dimer has little effect on tetramer assembly, as it does not cause repositioning of surface structures from both subunits that contribute to the dimer–dimer interface relative to each other (Figure 7). Thus, the diametrically opposed subunits that form the most extensive contacts at the centre of the tetramer, and whose active sites face each other (Figure 8), superimpose well with the equivalent subunits of a DmβUP tetramer. In contrast, the other two subunits at the more peripheral position, with active sites oriented towards the bulk solvent, are well aligned only on the site contributing to the interface, but show large shifts in equivalent Cα positions at the opposite site due to subunit rigid body rotation. The HsβUP tetramer is therefore somewhat flatter when viewed from “the top” as in Figure 7B (left). The interface contacts observed in the DmβUP crystal structure are, to a large extent, identical in HsβUP. For instance, from the 45 DmβUP residues involved in inter-dimer hydrogen bonds or salt bridges, only nine are not conserved in HsβUP, which includes six residues that form their dimer-linking hydrogen bonds via backbone functional groups. In comparison, the overall sequence identity of human and fruit fly βUP is “only” 64%.

To the surface features involved in interface formation belong also EL2 and EL3, which adopt conformations that are identical to those observed in DmβUP, except for that the tip of EL3 retains some flexibility. In the inner subunits involved in dimer–dimer interface formation, the last visible residue before the gap in density is H307, which was targeted in the here reported site-directed mutagenesis studies (Figure S8). Although the relatively low resolution of the cryo-EM derived density map does not allow a precise description of its interactions, it does indicate that H307 is indeed involved in hydrogen-bonding interactions with S300 and D309, as discussed earlier. In fact, the link with S300 gives EL3 the distinctive kink towards the protein surface required for dimer–dimer interface formation. Interestingly, in the two subunits whose active sites are facing outward, the EL3 loops are less well defined in the cryo-EM map (Figure S8). This can be explained with the contribution of different species (tetramers, hexamers, octamers) to the data from which the map is derived. It can be assumed that, similarly to DmβUP, in which all three entrance loops are disordered in the two subunits terminating the semi-circular homooctamer at both ends, the HsβUP entrance loops, and especially the most peripherally located EL3, will be ordered to differing degrees depending on whether or not they are located at dimer–dimer interfaces. That would not be the case for either peripheral loop set in tetrameric species, but one or both of these EL sets would be involved in dimer-dimer interactions in hexameric or octameric species, respectively (Figure 8).

Interestingly, the extent of density observed for the loop carrying the other interface mutation site, H173, also differs between “inner” and “outer” subunits. For the latter, density is lacking for the H173 residue itself and its immediate neighbours, indicating that also this surface region becomes more flexible when not interfacing with another dimeric unit. However, since the subunit C-termini are beyond residue R367 disordered in all subunits of the cryo-EM model, this is not due to lack of interaction of H173 with residues of an interfacing subunit, e.g., K374 (Figure S8).

Assuming that all dimer–dimer interfaces will be identical in higher oligomeric species, we created a model of the HsβUP octamer based on the tetramer structure obtained from the higher resolution cryo-EM map. Comparison of this model with the DmβUP crystal structure revealed that the above mentioned slight difference in subunit orientation and consequent “flattening” of the HsβUP tetramer would cause a wider diameter of the helical turn-shaped octamer (Figure S9). The increase in diameter is indeed also visible in the HsβUP octamer model obtained experimentally based on map **2**, but to a lesser extent. It thus seems that βUP oligomers are not rigid and can vary somewhat in helical pitch and diameter, possibly influenced by the environmental conditions.

## 4. Conclusions

The here reported site-directed mutagenesis data, inhibition studies, and tetrameric cryo-EM model of HsβUP obtained at low pH, which likely shares many features with the substrate-activated enzyme state at physiological pH, provided further insights into the mechanism of βUP activation. This allowed us to add detail to our model of allosteric regulation via oligomerization (Figure 8). It appears that enzyme activation is achieved by stable placement of the fourth residue of the catalytic tetrad, E207, in the active site. Since this glutamate belongs to one of the flexible loops shaping part of the active site and its entrance, this requires stabilization of the structures of these loops, which is achieved by their burial in dimer–dimer interfaces upon assembly of higher oligomers from inactive dimers. The active site remains accessible despite its partial occlusion by the entrance loops, allowing the activated state to be maintained throughout many catalytic cycles. An interesting feature of the mechanism is that the increase in catalytic activity may be solely achieved by an increase in the number of catalytically competent active sites brought about by enzyme oligomerization (Figure 8): EL stabilization cannot be achieved in dimeric species, and hence both active sites are catalytically incompetent, explaining the inactivity of all βUP mutants that lack the ability to oligomerize [14,15,24,44], including the H173, H307, and E207 mutants reported here. Assembly of two dimers to a tetramer makes two of the four active sites catalytically competent, whereas the other two are still “inactive”. Accordingly, a hexamer contains four competent active sites, and an octamer six, increasing the ratio of competent active sites in the different oligomers from 0%, via 50% and 67%, to 75%. Assembly of oligomers larger than the octamer would increase that ratio further, however, our cryo-EM (and negative stain EM) studies have not yet yielded unambiguous evidence that such higher oligomers exist. In contrast, homologous cyanide dihydratases and nitrilases can form long superhelices with similar architecture [45,46,47].

Our inhibition studies provided first clues regarding how NCβA and β-alanine binding may trigger enzyme association and dissociation, respectively. It appears that the ability or disability of enzyme ligands to engage in favourable interactions with F205 located on EL2 may distinguish activators from inhibitors. Additional studies are necessary to confirm this hypothesis. Furthermore, our site-directed mutagenesis results indicate that allosteric activation by the substrate NCβA requires residues able to donate two hydrogen bonds at positions 173 and 307, and a negative charge at position 207. At the cytosolic pH of 7.4, H173 and H307 would theoretically be more likely to be deprotonated than protonated. However, pKas of enzyme residues are influenced by their immediate neighbourhood, and that of both these histidines may thus be shifted to maintain a positively charged state at physiological pH. It is tempting to speculate that this may only occur upon binding of positive allosteric effectors such as the substrate NCβA, but again, further studies are required to obtain such insights. Nevertheless, the importance of the H173 and H307 charge state for the allosteric activation mechanism would explain why HsβUP is responsive to its allosteric effectors only within a relatively narrow window around physiological pH [24], and why allosteric regulation can be mimicked by shifts in pH. Further details of the allosteric mechanism may be revealed with structure determination of ligand bound states of HsβUP.

## Figures and Tables

**Table 1:** Cryo-EM data collection, refinement, and validation statistics.

Data Collection and Processing	
Microscope	Titan Krios G2
Nominal magnification	105,000
Voltage (kV)	300
Energy filter slit width (eV)	20
Defocus range (µM)	−0.6 to −2.0
Exposure time (s)	2.3
Dose rate (e^−^/px/s)	12.889
Total dose (e^−^/Å^2^)	43.661
Pixel size (Å)	0.824
Number of micrographs	10,512
Number of particles	
After 2D classification	603,157
After 3D classification	169,949
Symmetry	C2
Raw map resolution (Å) (FSC = 0.143)	3.33
Map sharpening B-factor	−109.9
**Refinement and Validation**	
Model composition	
Non-hydrogen atoms	11,438
Residues	1437
B-factor (Å^2^)	76.91
R.m.s.d.	
Bond lengths (Å)	0.002
Bond angles (°)	0.533
Molprobity score	2.33
Molprobity clash score	10.86
Rotamer outliers (%)	4.94
Ramachandran	
Overall Z-core (RMSD)	−0.51 (0.22)
Favoured (%)	96.34
Allowed (%)	3.10
Outliers (%)	0.56
Model-to-data fit	
CC(mask)	0.65
CC(volume)	0.67
CC(peaks)	0.61
PDB/EMDB ID	8PT4/17867

**Table 2:** Inflection temperatures of unfolding determined by thermal shift assays. Except for E207Q, measurements were performed at least in duplicate.

Variant	Inflection Temperatures (T_i_) [°C]
Wild-type	55.6 ± 0.6
C233S H173A	58.6 ± 0.1 54.0 ± 0.2
H173N	56.4 ± 0.9
E207Q	53.3
H307A	54.7 ± 0.4
H307N	54.3 ± 0.1

**Table 3:** Effect of substrate and product analogues on HsβUP enzymatic activity (at pH 6.5) and oligomer equilibrium (at pH 7.4). K_i_ values (assuming competitive inhibition) are given to provide an indication of the affinity for the compound.

Compound	Structure	K_i_ [µM]	Inhibition?	Effect on Oligomer Equilibrium
N-carbamoyl-β-alanine (NCβA)	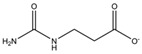	-	-	associative
isobutyric acid	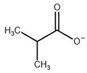	2269	yes	associative
y-aminobutyric acid (GABA) ^1^	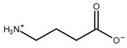	n.d. ^2^	yes	associative
5-amino levulinic acid ^1^	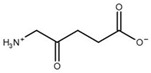	47	yes	associative
4-ureidobutyric acid ^1^	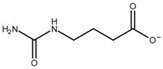	n.d. ^2^	yes	associative
β-aminoisobutyric acid (βAIBA) ^1^	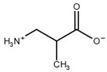	n.d. ^2^	yes	associative
β-alanine ^1^	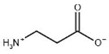	13	yes	dissociative
glycyl-glycine	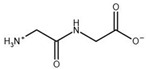	237	yes	dissociative
2-aminoisobutyric acid	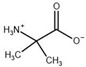	151	yes	dissociative
N-carbamoylglycine	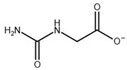	-	no	no effect
glutaric acid	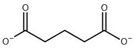	-	no	no effect

^1^ Data taken from [24]. ^2^ Not determined.

## Data Availability

The atomic coordinates and cryo-EM maps were deposited in the Protein Data Bank (PDB, accession code 8PT4) and in the Electron Microscopy Data Base (EMDB, Id: 17867).

## Supplementary Materials

The following supporting information can be downloaded at: https://www.mdpi.com/article/10.3390/biom13121763/s1, Figure S1: The reductive pyrimidine degradation pathway; Figure S2: Schematic of the interactions of H173 and H307 in the homology model of HsβUP and analysis of their pH dependency; Figure S3: Initial reaction rates of HsβUP wild type and variants; Figure S4: Kinetic data fit; Figure S5: Fourier shell correlation graphs; Figure S6: Local resolution of cryo-EM maps 1 and 2; Figure S7: Local correlation coefficient graphs; Figure S8: Cryo-EM map observed for the H173 and H307; Figure S9: Pairwise superposition of the HsβUP and DmβUP homooctamer models; Table S1: Primers used for the site-directed mutagenesis; Table S2: Goodness of fit according to competitive, uncompetitive, and non-competitive models for inhibition of HsβUP; Table S3: βUP structure comparison.
