# Supplementary material for: The Allosteric Regulation of Β-Ureidopropionase Depends on Fine-Tuned Stability of Active-Site Loops and Subunit Interfaces
Source: Biomolecules. 2023 Dec 8;13(12):1763. doi: 10.3390/biom13121763 (PMC10741476; doi:10.3390/biom13121763)
Supplement: Supplementary file 1 [file biomolecules-13-01763-s001.zip › biomolecules-2684552-supplementary.pdf]

biomolecules

Supplementary Materials

The Allosteric Regulation of Β-Ureidopropionase Depends on Fine-Tuned Stability of Active-Site Loops and Subunit Interfaces

Daniela Cederfelt [1], Dilip Badgujar [1,2], Ayan Au Musse [1,3], Bernhard Lohkamp [4], U. Helena Danielson [1] and Doreen Dobritzsch [1,*]

[1] Department of Chemistry—BMC, Uppsala University, 751 23 Uppsala, Sweden; daniela.cederfelt@kemi.uu.se (D.C.); dilip.badgujar@icm.uu.se (D.B.); helena.danielson@kemi.uu.se (U.H.D.)
[2] Department of Cell and Molecular Biology, Uppsala University, 751 23 Uppsala, Sweden
[3] School of Science and Technology, Örebro University, 701 82 Örebro, Sweden
[4] Department of Medical Biochemistry and Biophysics, Karolinska Institute, 171 77 Stockholm, Sweden; bernhard.lohkamp@ki.se
* Correspondence: doreen.dobritzsch@kemi.uu.se

Table S1. Primers used for the site-directed mutagenesis.

Variant	Forward primer 5' --> 3'	Reverse primer 3′ --> 5′
**C233S**	GGTCGTATTGCAGTGAATATTAGTTATGGTCGTCATCATC	GGATGATGACGACCATAACTAATATTCACTGCAATACGAC
**H173A**	TGATAGCGAAGCTGGTGATGTT	AACATCACCAGCTTCGCTATCA
**H173N**	CGTGATAGCGAAAATGGTGATGTTC	GAACATCACCATTTTCGCTATCACG
**H307A**	CAGCGGTGATGGTAAAAAAGCCGCTCAGGATTTTGGTTAT	ATAACCAAAATCCTGAGCGGCTTTTTTACCATCACCGCTG
**H307N**	CGGTGATGGTAAAAAAGCCAATCAGGATTTTGGTTATTTC	GAAATAACCAAAATCCTGATTGGCTTTTTTACCATCACCG
**E207Q**	GGGCGATTTTAACCAAAGCACCTATT	AATAGGTGCTTTGGTTAAAATCGCCC

Table S2. Goodness of fit according to competitive, uncompetitive and non-competitive models for inhibition of HsβUP.

Inhibitor	Inhibition model	Goodness of fit		
		Sum of Squares[1]	Sy.x[2]	K_M [μM]
Isobutyric acid	Competitive	14.1	0.5	179.9
	Uncompetitive	52.6	0.96	366.6
	Non-competitive	23.5	0.64	267.0
Glycyl-glycine	Competitive	38.3	0.92	174.8
	Uncompetitive	40.8	0.95	371.3
	Non-competitive	29.0	0.80	239.5
2-Aminoisobutyric acid	Competitive	36.0	0.90	175.0
	Uncompetitive	35.9	0.89	320.4
	Non-competitive	22.7	0.71	213.2

[1] Sum of squares $= \sum_{i=1}^{n}(x_i - \bar{x})^2$, with n = number of data points, x_i = value in a sample, $\bar{x}$ = sample mean $\bar{x}$. [2] $Sy.x = \sqrt{\frac{\Sigma(residual^2)}{n-K}}$, with residual = vertical distance (in y units) of the point from the fit line or curve, n = number of data points, K = number of parameters fit by regression.

Table S3. βUP structure comparison. Root-mean-square deviations (r.m.s.d., in Å) resulting from the superimposition of the individual subunits of the HsβUP cryo-EM structure with each other, and with the subunits of the HsβUP-T299C and DmβUP crystal structures. The r.m.d.s. for superposition of the entire HsβUP-T299C and DmβUP structures of are also given. The number of C_{α}-atoms aligned in each superimposition is stated in brackets. Grey shading indicates "central" subunits with closed active site entrance loops.

**HsβUP**	**A**	**B**	**C**	**D**
A	0			
B	0.002 (358)	0		
C	0.001 (358)	0.001 (358)	0	
D	0.054 (359)	0.053 (359)	0.001 (358)	0
**HsβUP-T299C**				
A	1.079 (300)	1.079 (300)	1.079 (300)	1.079 (300)
B	1.079 (300)	1.079 (300)	1.079 (300)	1.079 (300)
	Superposition as dimer		2.053 (591)	
**DmβUP**				
A	1.027 (326)	1.028 (326)	1.028 (326)	1.028 (326)
B	1.005 (351)	1.005 (351)	1.005 (351)	1.005 (351)
C	1.006 (353)	1.005 (353)	1.006 (353)	1.006 (353)
D	1.013 (351)	1.013 (351)	1.013 (351)	1.013 (351)
E	1.011 (352)	1.011 (352)	1.011 (352)	1.011 (352)
F	1.005 (351)	1.005 (351)	1.005 (351)	1.005 (351)
G	1.009 (353)	1.009 (353)	1.009 (353)	1.009 (353)
H	1.016 (320)	1.016 (320)	1.016 (320)	1.016 (320)
	Superposition as tetramer		2.252 (1286)	

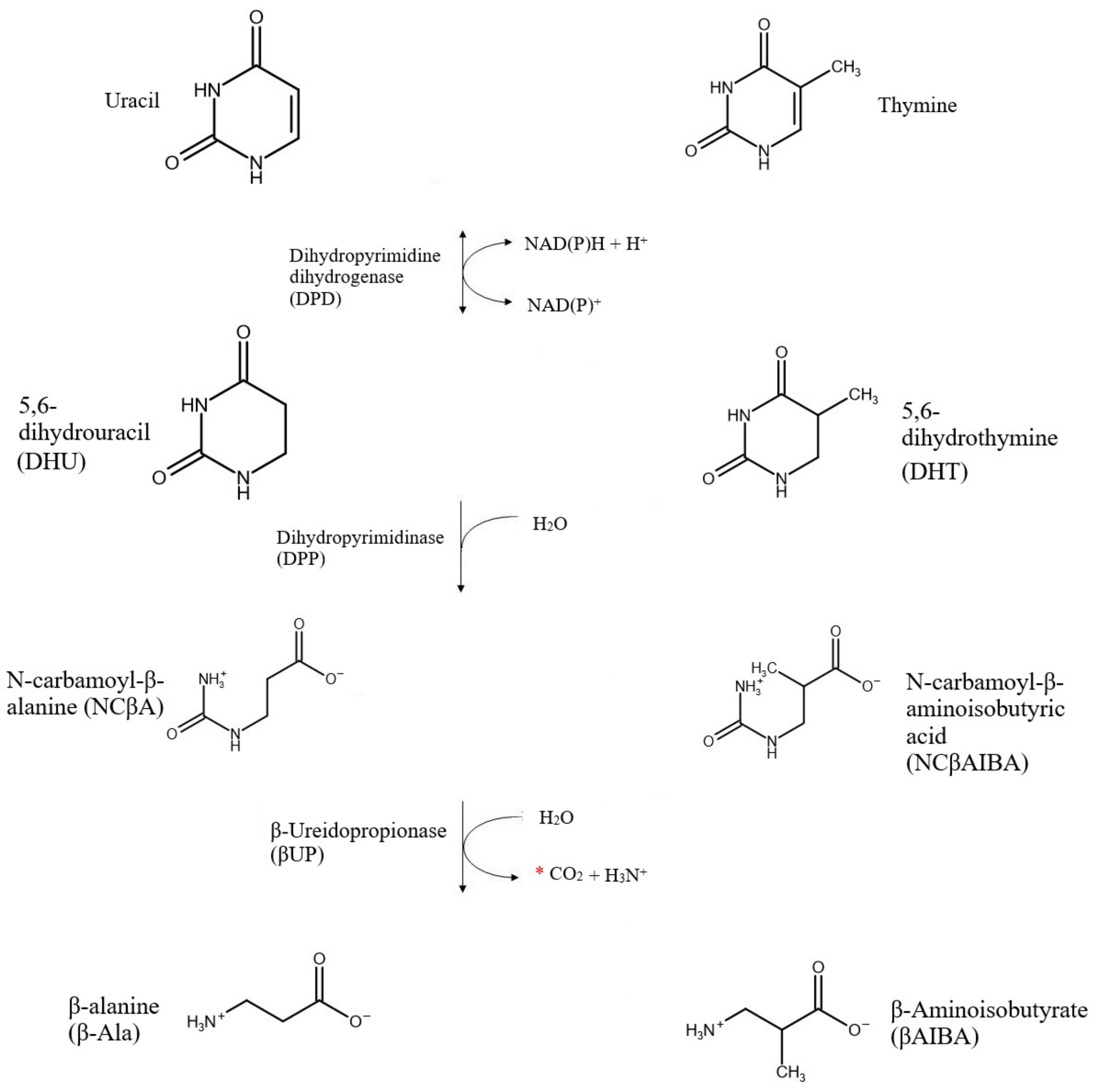

Figure S1. The reductive pyrimidine degradation pathway.

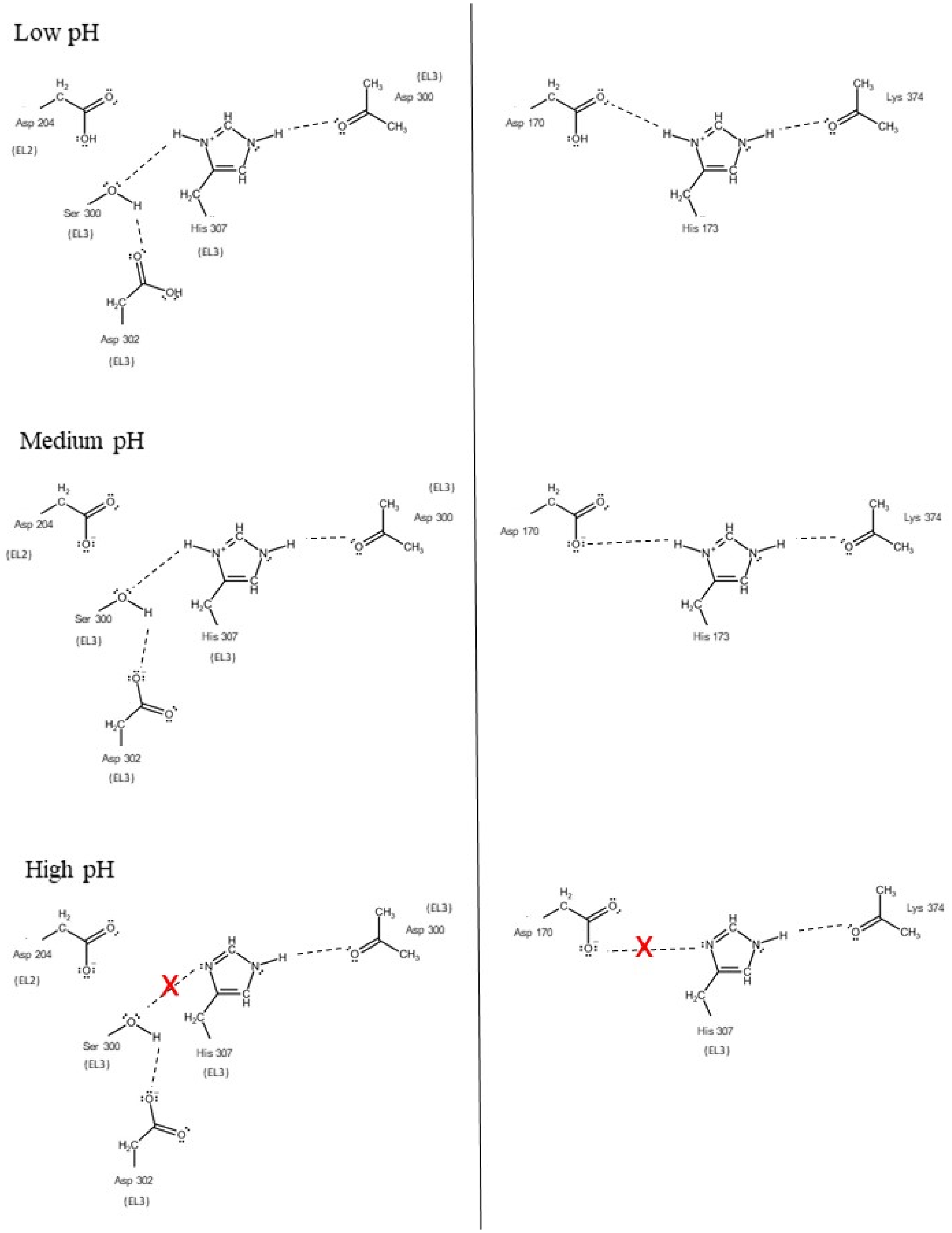

Figure S2. Schematic of the interactions of H173 and H307 in the homology model of HsβUP and analysis of their pH dependency. H173 as well as H307 are involved in two hydrogen bond interactions which require both of their ring nitrogen atoms to be protonated. Upon deprotonation of the residues at high pH, one of the interactions should be broken.

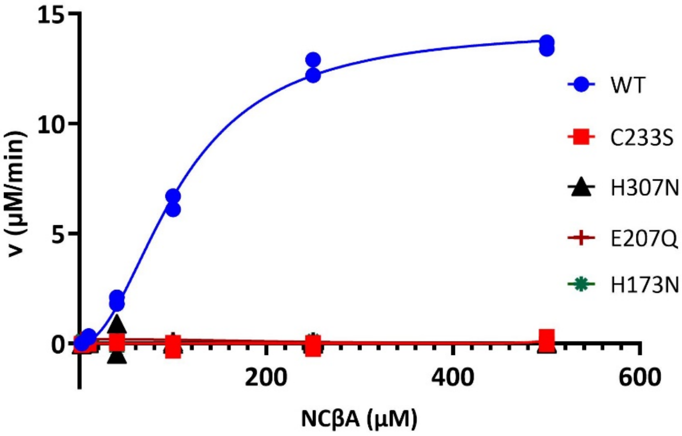

Figure S3. Initial reaction rates of HsβUP wild-type and variants as a function of NCβA concentration at the pH of optimal catalytic activity (6.5) and 37°C. The data points for both measurements performed per variant are shown. The data obtained for H307A and H173A correspond to those shown for their counterparts H307N and H173N.

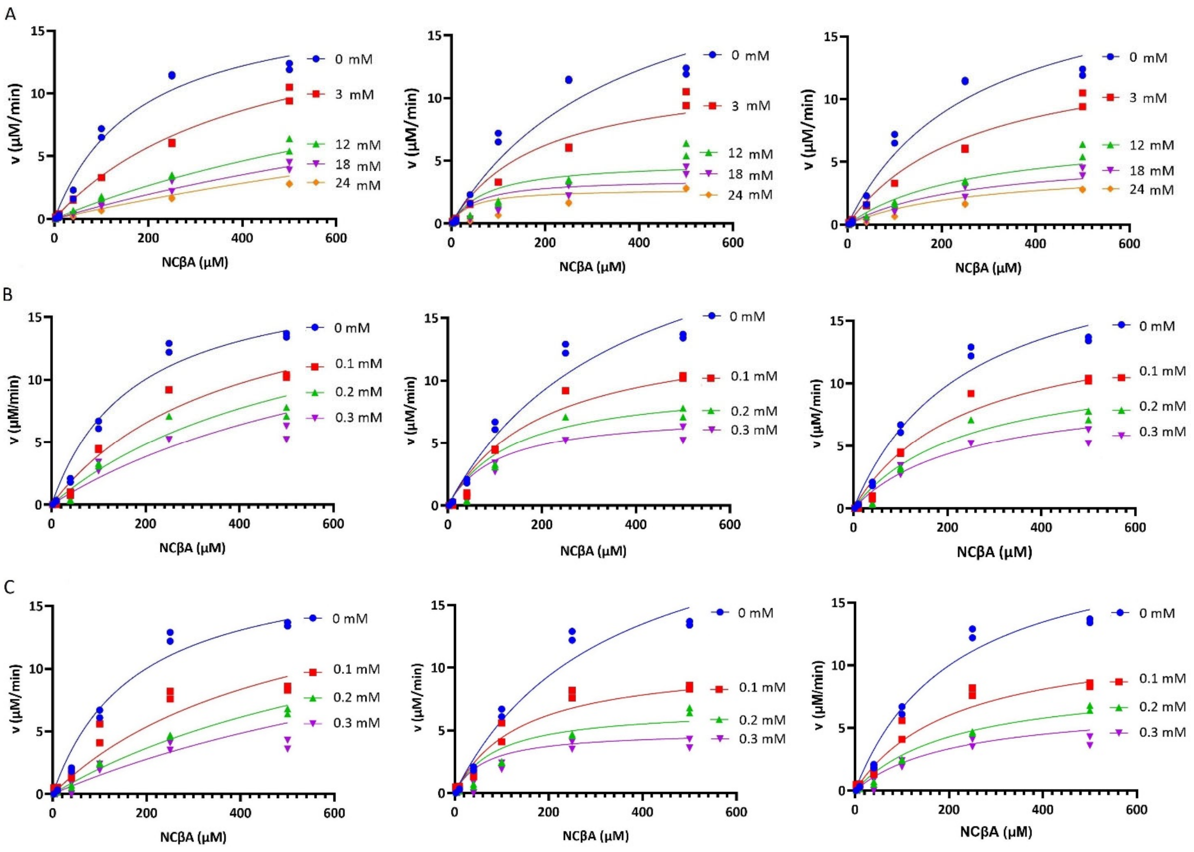

Figure S4. Kinetic data fit. HsβUP reaction rates obtained at varying substrate concentrations and in presence of (A) isobutyric acid, (B) glycyl-glycine, and (C) 2-aminoisobutyric acid were fitted assuming classic competitive (left), uncompetitive (middle), and non-competitive inhibition modes. Goodness of fit values are given in Table S2.

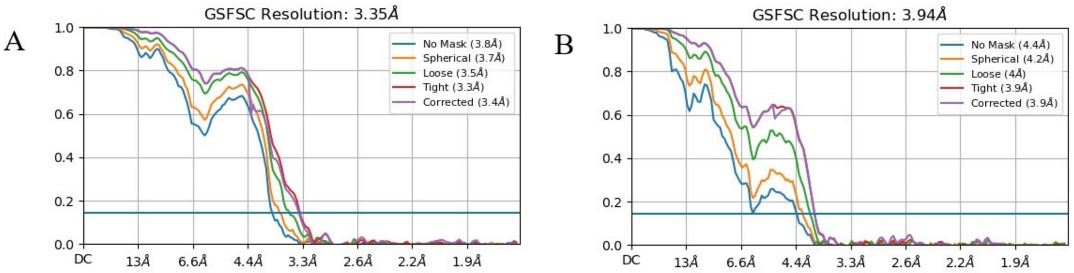

Figure S5. Fourier shell correlation graphs. The Gold-standard Fourier shell correlation (GSFSC) graphs for cryo-EM maps 1 (A) and 2 (B), obtained from non-uniform refinement in cryoSPARC [38, 39].

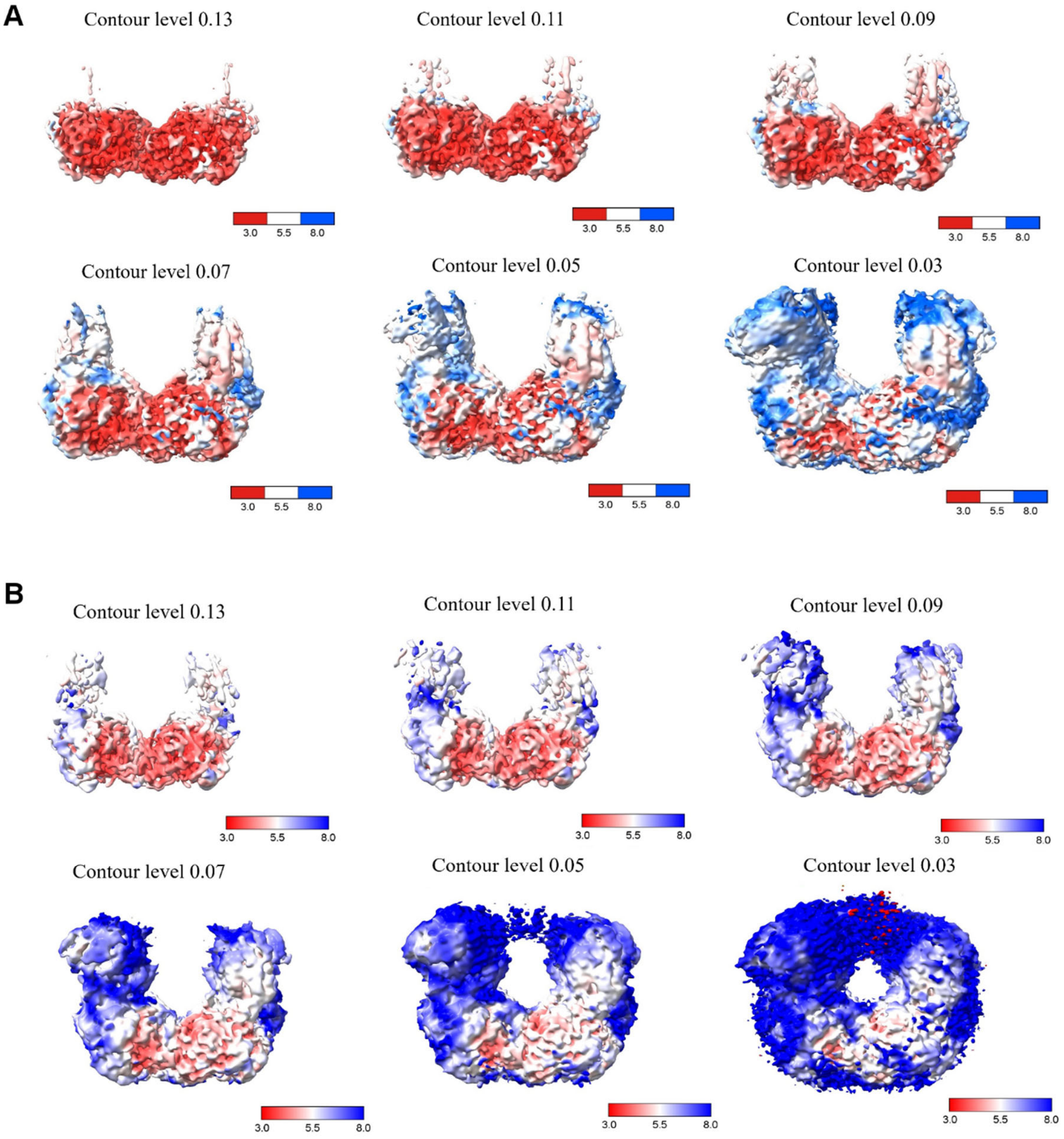

Figure S6. Local resolution of cryo-EM maps 1 (A) and 2 (B). The maps are shown at the indicated contour levels and coloured according to local resolution from highest resolution (red) to lowest resolution (blue).

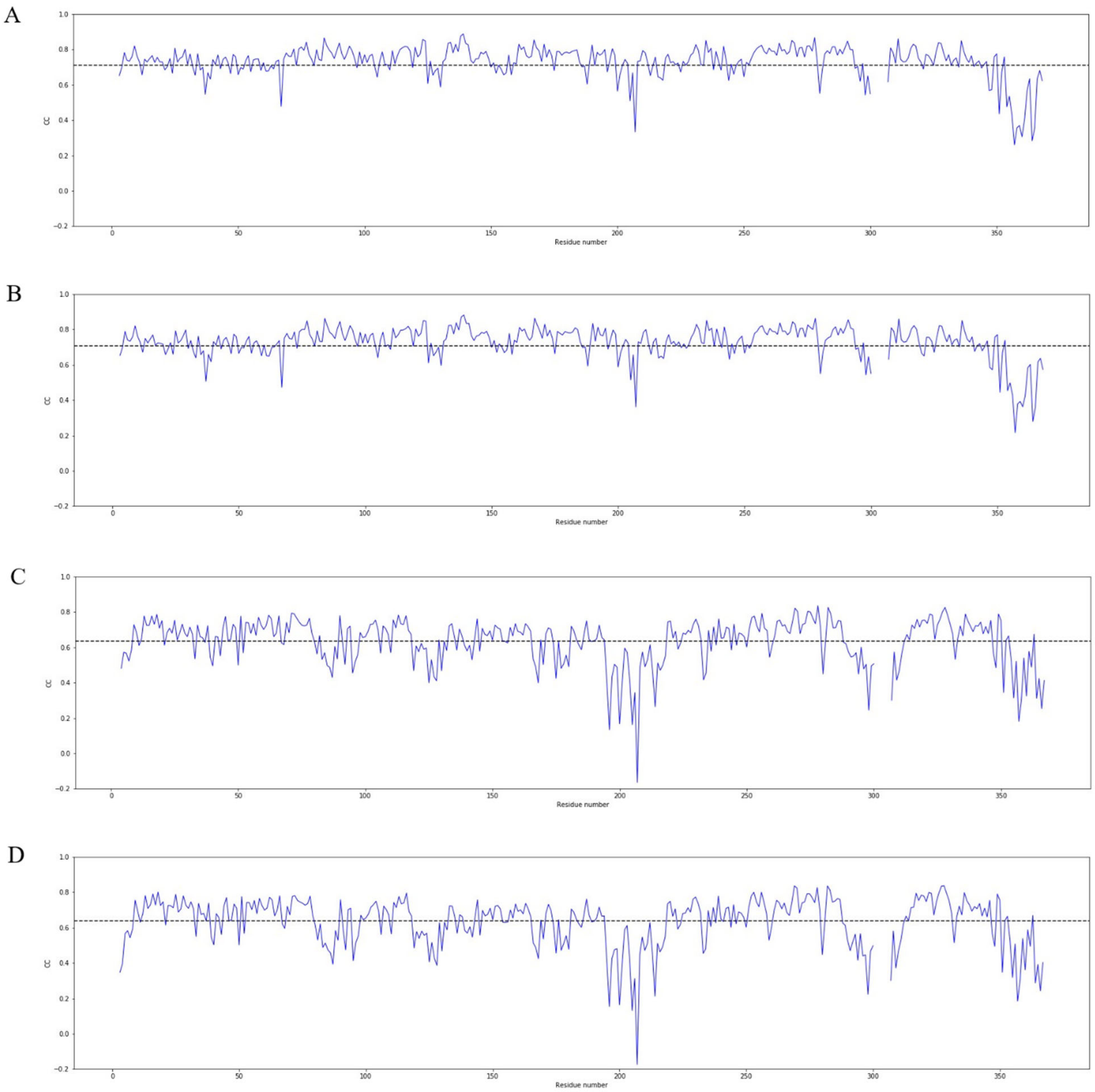

Figure S7. Local correlation coefficients of model-to-map fitting obtained for chains A, B, C, and D after model refinement.

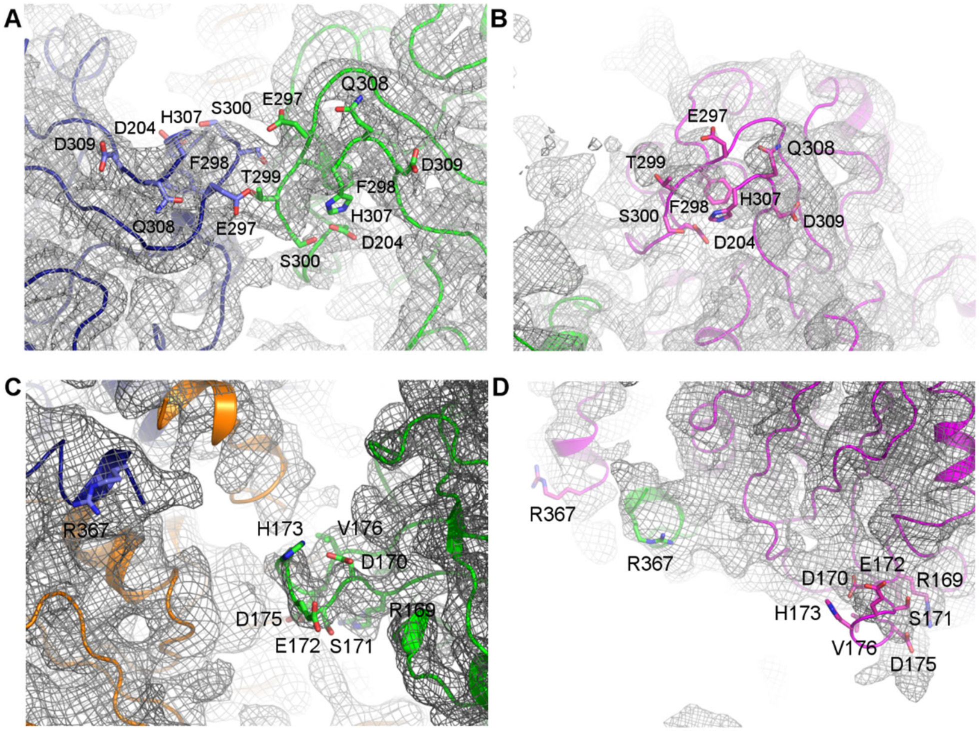

Figure S8. Cryo-EM map (map **1**) observed for the H173 and H307. The four subunits of the deposited cryo-EM structure are shown as cartoons coloured blue (chain A), green (chain B), magenta (chain C) and orange (chain D), respectively, with chains A and B representing the "inner" subunits forming the most extensive contacts at the dimer-dimer interface. H173, H307, and surrounding or interacting residues are shown as sticks with correspondingly coloured carbon atoms. (**A**) At the dimer-dimer interface, density is observed for most of the EL3 carrying H307, except residues 301-306. (**B**) In the "outer" subunits (chains C, D), EL3 is not in contact with a neighbouring subunit, and thus more flexible, as indicated by the lack or less well defined density for residues 297-307. (**C**) In chains A and B representing the "inner" subunits, H173 is located close to subunits from a neighbouring dimer. The lack of density for a large part of the C-terminal helix (identified by the side chain of R367) and the following loop indicates that H173 does not make stable contacts to these elements. Nevertheless, the entire loop carrying H173 is resolved in the density map. (**D**) In the "outer" subunits, the corresponding loop is fully solvent-exposed, causing its increased flexibility indicated by the lack of density for H173 and neighbouring residues.

A

B

C

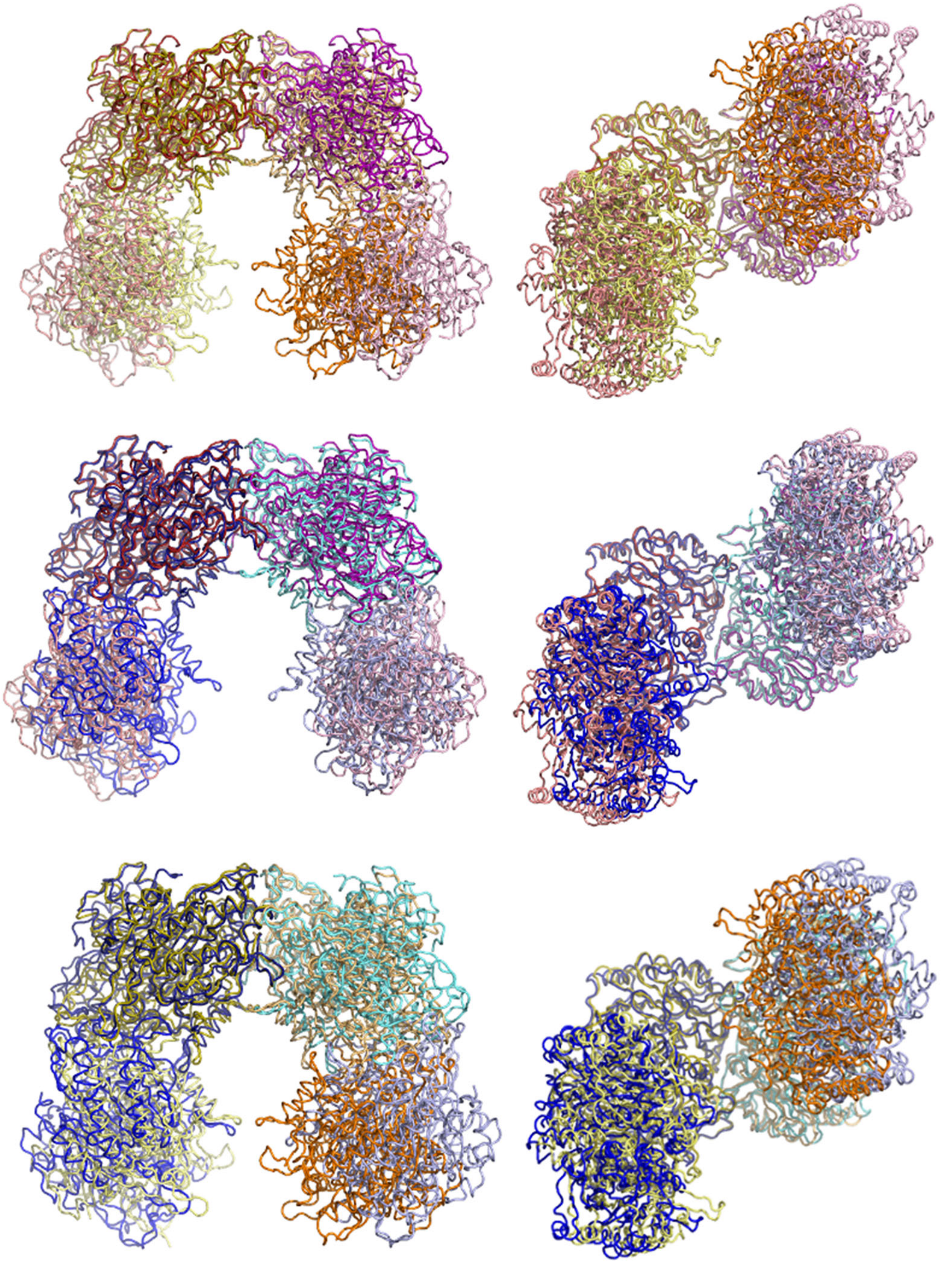

Figure S9. Pairwise superposition of the HsβUP and DmβUP homooctamer models. Shown are two orientations that are related to each other by a ~90° rotation around an axis lying horizontally in the paper plane. The four dimeric units forming the homooctamer of the DmβUP crystal structure are coloured in different yellow and orange shades, those of the HsβUP homooctameric lower resolution cryo-EM structure in different blue shades, respectively. An artificial model of the HsβUP homooctamer constructed using the same interface geometry as observed between the two dimeric units in the homotetrameric higher resolution cryo-EM model is coloured in pink and red shades. The superposition was based on one of the "inner" subunits. (**A**) Superposition of the artificial HsβUP cryo-EM model with the DmβUP crystal structure. (**B**) Superposition of the artificial with the experimentally observed HsβUP cryo-EM structure. (**C**) Superposition of the experimentally observed HsβUP cryo-EM structure with the DmβUP crystal structure.
